# Rapid body colouration changes in *Oryzias celebensis* as a social signal influenced by environmental background

**DOI:** 10.1098/rsbl.2024.0159

**Published:** 2024-07-24

**Authors:** Ryutaro Ueda, Satoshi Ansai, Hideaki Takeuchi

**Affiliations:** ^1^ Graduate School of Life Sciences, Tohoku University, Sendai, Miyagi 980-8577, Japan; ^2^ Laboratory of Genome Editing Breeding, Graduate School of Agriculture, Kyoto University, Kyoto 606-8507, Japan

**Keywords:** *Oryzias celebensis*, rapid colouration change, camouflage, social signal, co-option, visual signal

## Abstract

Rapid body colouration changes in some animals, such as chameleons and octopuses, serve dual functions: camouflage and intraspecific communication. It has been hypothesized that these colouration changes originally evolved to provide camouflage and subsequently were co-opted as social signals; however, experimental model systems that are suitable for studying such evolutionary processes are limited. Here, we investigated the relationship between rapid colouration changes of the blackened markings and aggressive behaviours in male *Oryzias celebensis*, an Indonesian medaka fish, under triadic relationships (two males and one female) or three males conditions with two different environmental backgrounds. In an algae-covered tank, mimicking the common laboratory rearing conditions, males with blackened markings exhibited more frequent attacks towards different conspecific individuals compared with non-blackened males and females. The blackened males were seldom attacked by non-blackened males and females. By contrast, neither aggressive behaviours nor black colouration changes were observed in the transparent background condition with a brighter environment. These indicated that the blackened markings in *O. celebensis* serve as a social signal depending on the environmental backgrounds. Considering that such colouration changes for camouflage are widely conserved among teleost fishes, the traits are likely to be co-opted for displaying social signals in *O. celebensis*.

## 1. Introduction

Body colouration of animals serve multiple functions, such as camouflage to avoid predation, mate attraction and rival deterrence [1]. Conspicuous body colouration acting as social signals for intraspecific communication are often observed in males and are therefore thought to have been evolved under sexual selection, whereas these conspicuous colourations could be disadvantageous under natural selection, as the colourations could increase the predation pressure. This trade-off between sexual and natural selection could drive the evolution and diversification of animal body colouration [2,3]. One of the possible mechanisms for expressing such conspicuous colourations with reducing the risk of predation is a highly plastic change of the body colouration [4]. For example, although chameleons are a well-known species group that can change their body colouration extremely rapidly within seconds in response to changes in their environmental background for camouflage, some chameleon species are also known to use the colouration change as a social signal for the intraspecific communication [5,6]. Given the evolutionary patterns in the family Chamaeleonidae, the rapid and plastic colouration changes originally evolved to reduce predation pressures by camouflage and were subsequently co-opted as social signals [5]. However, experimental model animals that are suitable for studying such evolutionary processes remain limited.

**Figure 1.**
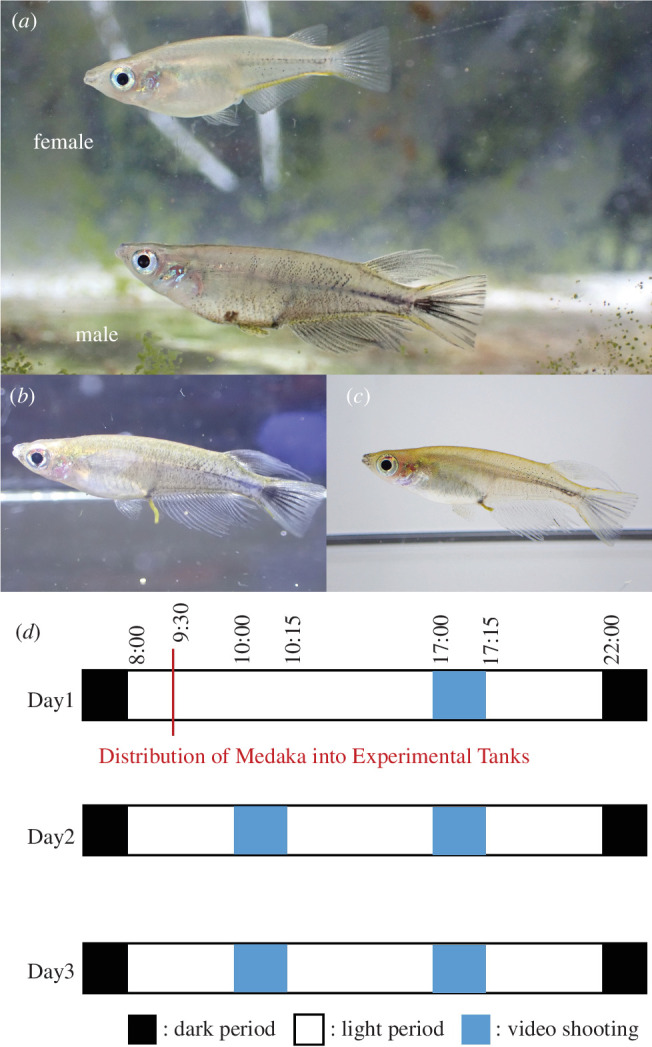
Body colouration changes in *Oryzias celebensis*. (*a*) Representative images of female and male *O. celebensis*. Some male *O. celebensis* exhibit distinctive blackened markings on their fins and sides. (*b,c*) The blackened markings can change rapidly within a minute when shifting black and white backgrounds ((*b*) and (*c*) denote the same individual). (*d*) Timetable for the triadic behavioural assays.

To address this issue, we used an Indonesian medaka fish, *Oryzias celebensis* as an experimental organism. Medaka fish (family Adrianichthyidae) are widely distributed in East and Southeast Asia, and 20 of the 39 species are endemic to Sulawesi, Indonesia [7,8]. These fish exhibit significant diversification in sexually dimorphic traits such as body colourations that are prominently displayed in males, making them an excellent model for exploring the evolutionary genetic mechanisms underlying sexual dimorphism [9]. Among these endemic species, *O. celebensis* males exhibit distinctive blackened markings on their fins and sides (figure 1*a*), and the colouration changes rapidly within a minute (electronic supplementary material, video S1). We have observed that such colouration changes can be rapidly induced by altering their environmental background conditions from black to white or white to black, without any social context (figure 1*b,c*). Additionally, we observed that under group-housing conditions in the laboratory, the number of individuals with blackened markings tended to increase at higher rearing densities. This observation led us to predict that intra-species competition may induce the blackening of some individuals. Therefore, we hypothesized that the body colouration changes in *O. celebensis* serve dual functions: camouflage and social signalling. Several species of teleost fishes, including the Japanese medaka, have been observed to exhibit background adaptation [10,11]. Given the validity of this hypothesis, these fish could potentially be an excellent model for exploring the evolutionary processes of the co-option of camouflage traits as social signals. Here, we established a behavioural experimental paradigm that allows consistent observation of aggressive behaviours with stable monitoring of their body colouration changes. Using this behavioural paradigm, we investigated the relationship between aggressive behaviours and body colouration changes, as well as the effects of environmental backgrounds.

## 2. Results

**Figure 2.**
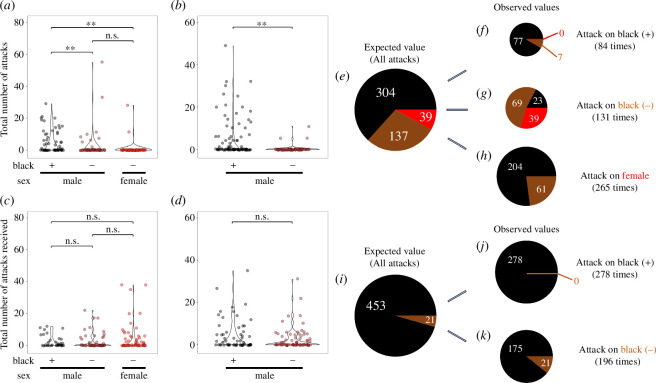
Relationship between attacks and male body colouration. (*a,b*) Number of attacks under the triadic (two males and a female) (*a*) and three males (*b*) conditions. The number of attacks by males with blackened markings was much higher than that by males without blackened markings and females. (*c,b*) The number of attacks received under the triadic (*c*) and three males (*d*) conditions. The number of attacks received did not differ significantly between conditions. *p < 0.05, **p < 0.01, and not significant (n.s.) according to generalized linear mixed models followed by Tukey’s post hoc test. (*e–k*) Pie charts showing the directions of attack events under the triadic (*e–h*) and three males (*i–k*) conditions, (*e,i*) total number of attacks, (*f, j*) attacks on males with blackened markings, (*g,k*) attacks on males without blackened markings, and (*h*) attacks on females. In each case, the left pie chart represents the total number of attacks, while the right pie charts show the division of the left pie chart based on the direction of the attacks. Black, brown and red slices represent males with blackened markings, males without blackened markings, and females, respectively. The size of each slice reflects the number of attacks received.

To determine whether body colouration correlates with attack frequency, we examined the numbers of attacks and the patterns of body colouration changes in small tanks under the following conditions: an algae-covered tank containing two males and one female (triadic) (*n* = 13); an algae-covered tank containing three males (*n* = 12). We recorded the number of attacks and the associated body colouration during these attack events for each individual under the triadic condition. Males with blackened markings exhibited a significantly higher number of attacks compared with males without blackened markings and females (generalized linear mixed model (GLMM)) followed by Tukey’s post hoc test: black(+) versus black(−), estimate = 2.49, standard errors (s.e.) = 0.952, *p* = 0.0244; black(+) versus female, estimate = 4.89, s.e. = 1.122, *p* < 0.0001; figure 2*a*). Similar tendencies were found in the three males condition (GLMM followed by Tukey’s post hoc test: black(+) versus black(−), estimate = 4.88, s.e. = 1.01, *p* < 0.0001; figure 2*b*). These findings indicate that the *O. celebensis* males with blackened markings exhibited higher aggression towards different conspecific individuals. To determine whether the susceptibility to attacks varies with body colouration, we recorded the number of attacks each individual received and their body colouration at the time of the attack in the triadic condition. The number of attacks received did not differ significantly in relation to body colouration (GLMM followed by Tukey’s post hoc test: black(+) versus black(−), estimate = 0.378, s.e. = 0.574, *p* = 0.788; black(+) versus female, estimate = 0.0811, s.e. = 0.473, *p* = 0.984; black(−) versus female, estimate = −0.296, s.e. = 0.474, *p* = 0.806; figure 2*c*). No significant difference was found between the males with and without blackened markings in the number of attacks received under the three males condition (GLMM followed by Tukey’s post hoc test: black(+) versus black(−), estimate = 0.132, s.e. = 0.361, *p* = 0.715; figure 2*d*).

Next, to examine whether there are biases in the body colouration of the individuals attacked, we analysed the directions of the attack events. In the case of the triadic condition, the observed attack values on males with blackened markings (figure 2*f*), males without blackened markings (figure 2*g*) and females (figure 2*h*) differed significantly from the expected values (figure 2*e*) (chi-square test: attacks on males with blackened markings: *χ^2^*_₂,₈₄_ = 29.491, *p* < 0.0001; attacks on males without blackened markings: *χ^2^*_₂,₁₃₁_ = 145.61, *p* < 0.0001; attacks on females: *χ^2^*_₁,₂₆₅_ = 8.0128, *p* = 0.004645). Under the three males condition, the number of attacks on males with blackened markings (figure 2*j*) and males without blackened markings (figure 2*k*) also differed significantly differently from the expected value (figure 2*i*) (chi-square test: attacks on males with blackened markings: *χ^2^*_₁,₂₇₈_ = 12.887, *p* = 0.0003308; attacks on males without blackened markings: *χ^2^*_₁,₁₉₆_ = 18.279, *p* < 0.0001). These findings demonstrated that males with blackened markings were predominantly targeted by other males with blackened markings, while attacks from males without blackened markings or females were rare. On the other hand, males without blackened markings were attacked not only by males with blackened markings but also by other males without blackened markings and females. Additionally, females under the triadic condition received a similar attack frequency as males.

**Figure 3.**
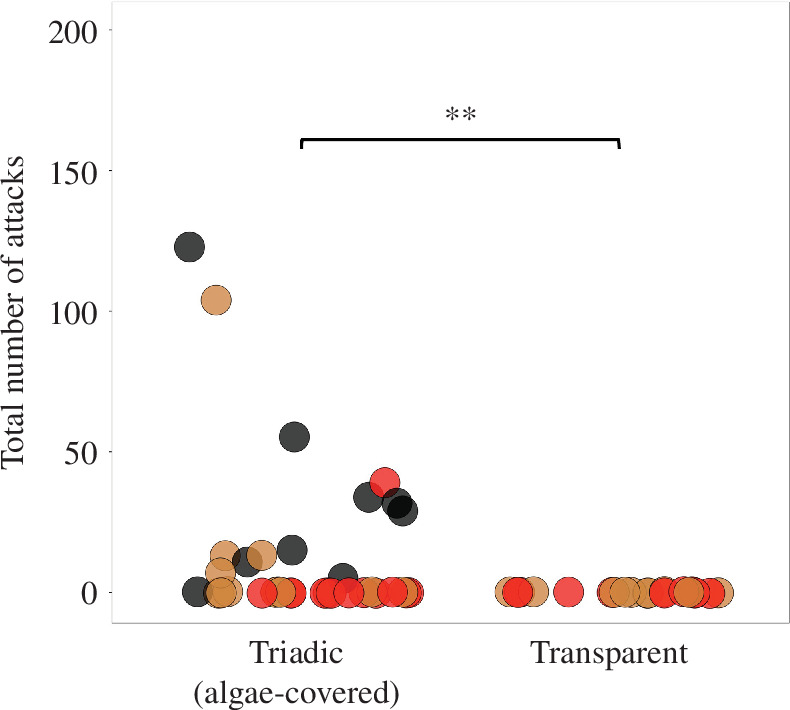
The effect of environmental backgrounds on aggressive behaviours. The total number of attacks in each test was counted under the triadic condition in test tanks covered with algae and in the transparent tanks. The black, brown and red points represent males with blackened markings, males without blackened markings and females, respectively. Neither aggressive behaviours nor black colouration changes were observed under the transparent condition. **p < 0.01 according to Mann–Whitney *U*-test.

Finally, to investigate the effect of experimental backgrounds on aggressive behaviours and body colouration changes in *O. celebensis*, we examined the numbers of attacks and the patterns of body colouration changes in small tanks under the condition: a transparent tank with no algae containing two males and one female (transparent) (*n* = 10). As mentioned previously, males with blackened markings and aggressive behaviours were consistently observed in the algae-covered condition. By contrast, neither aggressive behaviours nor black colouration changes were observed under the transparent condition (Mann–Whitney *U*-test: triadic (algae-covered) versus transparent, *Z* = 3.1052, *p* = 0.001905; figures 1*b,c* and 3), indicating that the algae-covered walls of the tank were required for the emergence of both the attacks and the black colouration changes.

## 3. Discussion

The findings of this study demonstrated that male *O. celebensi*s with blackened body markings exhibited higher levels of aggression towards other members of the same species. In many animal species, limited resources such as food, territory and mates can drive intraspecific competition for their access [12]. The fact that females were often attacked by males in the triadic condition suggests that the aggressive behaviour observed in this study may be owing to competition for resources such as food rather than from male-to-male competition for mates [13]. In the context of these intraspecific competitions, non-physical aggressive displays can prevent escalation to physical contact [14,15]. Consistently, the blackened markings observed in *O. celebensis* males could deter attacks from males without blackened markings and females (figure 2*e–k*). In some animal species, visual threat signals, which are specific behavioural patterns, indicate aggressive motivation to assist in conflict resolution [16–18]. Our findings suggest that the blackened markings in *O. celebensis* males may act as visual cues to signal dominance and fighting ability, thereby facilitating the resolution of resource disrupts.

This study demonstrated that aggressive behaviours and the presence of males with blackened markings were consistently observed in the algae-covered tanks, whereas aggressive displays and colouration changes were notably absent in the transparent tanks. There are two possible explanations for these phenomena. The first possibility is that the transparent background might increase stress and fear in the males thereby suppressing their aggression while simultaneously influencing their body colouration. The second possibility is that visual input from the background directly regulates both body colouration and aggression through a common neural network. Similar examples of background influencing both body colouration changes and aggressive behaviours have been reported in other fish species. In the Arctic char (*Salvelinus alpinus*), in contrast to *O. celebensis*, darker body colouration serves as a signal of social subordination [19]. On a white background, aggressive behaviours between males were increased, and subordinate males became darker than dominant ones, whereas on a black background, aggressive behaviours between males were significantly reduced [19]. The rainbowfish (*Melanotaenia australis*) also exhibited darker body colouration in subordinate males [20]. Compared with males that did not darken, males that darkened for camouflage received increased aggression from dominant males [20]. Further research is needed to investigate whether the neural mechanisms involved in camouflage and the regulation of aggression are independent or shared.

Background adaptation, a type of camouflage characterized by changes in body colouration in response to background, is largely conserved in different fish species [21]. These physiological responses are known to be caused by the translocation of pigment granules within the chromatophores in the skin [22]. By contrast, the use of the blackened colouration change as social signals has only been observed in the specific lineages [19,20], suggesting that the traits have been used widely for the background adaptation and later been co-opted for intraspecific communication in the specific lineages including *O. celebensis*. The availability of reference genome assemblies and genome editing techniques in *O. celebensis* and other Indonesian medaka fishes [23] will provide a new avenue for investigating the detailed molecular mechanisms of how the background adaptation traits have been co-opted for social signals.

## 4. Material and methods

### (a) Fish and housing conditions

*Oryzias celebensis* (the Ujung pandang strain) was provided by the National Bioresource Project (NBRP) medaka (RS278; https://shigen.nig.ac.jp/medaka/). Fish were maintained in groups in a glass tank (60 cm × 30 cm × 36 cm (height)) containing approximately 30–40 individuals with a roughly 1 : 1 male-to-female ratio and fed nauplii of brine shrimp or powdered food once a day between 12.00 and 13.00. All fish were hatched and bred in our laboratory. Sexually matured male and female fish 3−15 months of age were subjected to behavioural trials. The water temperature was approximately 29°C and light was provided by LED lights for 14 h per day (08.00−22.00).

### (b) Behavioural trials

We observed aggressive behaviours among three adult fish in an acrylic tank (24 cm × 14 cm × 15 cm) for three consecutive days (figure 1*d*) under the following three conditions: the triadic condition consisting of two males and one female in each tank covered with algae on the wall, the three males condition consisting of three males in each tank covered with algae on the wall, and the transparent condition consisting of two males and one female in each transparent tank without algae on the wall. We used the same set of adult fish for all three conditions, which were housed for 2 or more days under the conditions described above. A video of each behavioural trial was recorded twice a day at 10:00 (morning) and 17.00 (evening) using a digital camera (Go Pro Hero9 Black) (figure 1*c*). In the first morning during the assay (between 09.30 and 10.00), test fish were randomly transferred from the aquarium to each experimental tank. Fifteen minutes after starting the video recording, the test fish were transferred to a small bag with a zip-lock closure, and then photos of the whole body of the test fish were taken using a digital camera (TG-6, Olympus) for individual identification by their fin shapes and pigmentation patterns of the body surface. After obtaining the photo, each fish was returned to their experimental tank. In the first 15 min of the video recordings, we defined aggressive behaviours and their targets based on previous studies on the aggressive behaviour of Japanese medaka (*Oryzias latipes*) [24,25]. Specifically, we defined aggressive behaviours as a rapid swim towards another individual, with the fish receiving this behaviour fleeing in response. The target is defined as the individual being approached. We also described the direction of each attack event and then determined the body colouration of each test fish while it attacked or was attacked by the other fish, using still images from videos and comparing them to the representative images of males with black markings (figure 1*a,b*). In this study, a male whose markings blackened at least once in each video recording was considered as a male with blackened markings.

### (c) Statistical analysis

To examine whether attack frequencies and attack susceptibility vary based on body colouration, we compared the body colouration with the number of attacks performed and the number of attacks received. We used GLMM with negative binomial distributions and a log link function, employing the ‘glmmTMB’ function in the package *glmmTMB* v. 1.1.7 [26] in R v. 4.2.3. Body colouration of each individual while attacking or being attacked by other individuals was included as a fixed factor, and experiment tank numbers were included as random factors. We included the combination of the three fish as a random factor in our models. For a post hoc test, *p*-values adjusted with Tukey’s method were calculated using the package *emmeans* v. 1.8.5 [27]. Furthermore, to investigate the effect of experimental backgrounds on aggressive behaviours and body colouration changes, we conducted a Mann–Whitney *U*-test using the ‘wilcox_test’ function in the *coin* package v. 1.4.3 implemented in R v. 4.2.3 [28].

Next, we investigated the potential biases (preferences) in the direction of aggressive behaviour based on body colouration. Specifically, we examined whether individuals with certain body colourations are more likely to be attacked by (or less likely to be attacked by) individuals with specific body colouration. Attack direction indicates which individuals with certain body colourations attacked (or were attacked by) individuals with specific body colouration. We analysed the total number of attacks for each body colouration and analysed the directions of attack events, identifying which colouration was the attacker and which was the attack target in each interaction. We set the total ratio of the total number of attacks as an expected value, and the ratio of the total number of attacks categorized by the directions of attack events as observed values. Significant differences between the observed values and expected ones were analysed using the chi-square test implemented in R v. 4.2.3.

## Data Availability

Data are available from the Dryad Digital Repository [29]. Supplementary material is available online [30].
